# Age-Associated Loss in Renal Nestin-Positive Progenitor Cells

**DOI:** 10.3390/ijms231911015

**Published:** 2022-09-20

**Authors:** Marina I. Buyan, Nadezda V. Andrianova, Vasily A. Popkov, Ljubava D. Zorova, Irina B. Pevzner, Denis N. Silachev, Dmitry B. Zorov, Egor Y. Plotnikov

**Affiliations:** 1 Faculty of Bioengineering and Bioinformatics, Lomonosov Moscow State University, 119992 Moscow, Russia; 2 A.N. Belozersky Institute of Physico-Chemical Biology, Lomonosov Moscow State University, 119991 Moscow, Russia; 3 V.I. Kulakov National Medical Research Center of Obstetrics, Gynecology and Perinatology, 117997 Moscow, Russia

**Keywords:** stem cells, kidney regeneration, S3-segment, papilla, mitochondrial membrane potential, oxygen–glucose deprivation, cisplatin

## Abstract

The decrease in the number of resident progenitor cells with age was shown for several organs. Such a loss is associated with a decline in regenerative capacity and a greater vulnerability of organs to injury. However, experiments evaluating the number of progenitor cells in the kidney during aging have not been performed until recently. Our study tried to address the change in the number of renal progenitor cells with age. Experiments were carried out on young and old transgenic nestin-green fluorescent protein (GFP) reporter mice, since nestin is suggested to be one of the markers of progenitor cells. We found that nestin^+^ cells in kidney tissue were located in the putative niches of resident renal progenitor cells. Evaluation of the amount of nestin^+^ cells in the kidneys of different ages revealed a multifold decrease in the levels of nestin^+^ cells in old mice. In vitro experiments on primary cultures of renal tubular cells showed that all cells including nestin^+^ cells from old mice had a lower proliferation rate. Moreover, the resistance to damaging factors was reduced in cells obtained from old mice. Our data indicate the loss of resident progenitor cells in kidneys and a decrease in renal cells proliferative capacity with aging.

## 1. Introduction

Aging affects most of the organism’s functions, with a well-documented deteriorating impact on health. Kidneys, like other organs, undergo physiological changes with age, impairing many essential renal functions [1]. Kidney aging is associated with the loss of both glomeruli and tubules. In addition to nephron loss, glomerulosclerosis and tubulointerstitial fibrosis are observed in the old kidneys, which were proposed to serve as compensatory mechanisms for replacing lost structures [2]. Decrease in kidney function as a result of acute kidney injury (AKI) occurs mainly in elderly people [3]. Moreover, the kidneys of old people are more susceptible to damage [4], and AKI in such patients is more likely to transform into chronic kidney disease (CKD) [5].

One of the possible reasons for the greater vulnerability of old tissues to injury may be a diminished number of progenitor cells. Stem cells depletion and cellular senescence are believed to be one of the main hallmarks of aging [6]. The reduced number of progenitor cells has been well documented for the muscles, revealing the age-related loss of satellite cells and development of sarcopenia [7], as well as for the resident progenitor cells of the brain and intestine [8,9,10]. However, such experiments evaluating the number of renal progenitor cells during aging have not been performed before [11]. To our knowledge, only a single study showed that the number of bromodeoxyuridine (BrdU)-labeled cells diminished during aging [12], and experiments explaining the decrease in the number of cells carrying any markers of stemness have not been carried out until now. Therefore, the dynamics of renal stem/progenitor cell content with age needs to be investigated.

**Figure 1:**
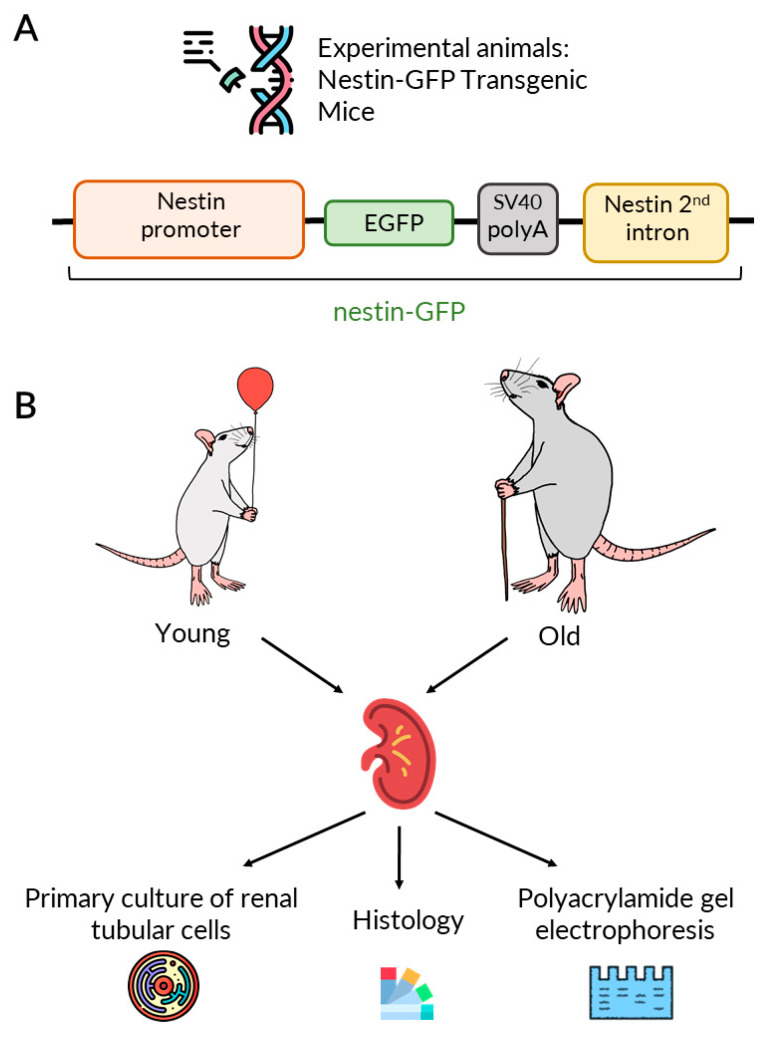
Experimental design. (**A**) Scheme of the nestin-GFP construct of transgenic nestin-GFP mice. For the details see [13]. (**B**) The experimental design of our study.

To study this issue, we used transgenic nestin-green fluorescent protein (GFP) reporter mice (Figure 1) [13]. Nestin is considered a marker of progenitor cells in different organs since it is expressed in the neuronal progenitor cells and oligodendrocytes, in the germ skeletal and cardiac myocytes, and in the mesonephros and endothelial cells of developing vessels [14]. In human kidneys, it has been shown that CD133^+^ cells, considered as progenitor cells, coexpress nestin [15]. Earlier, nestin^+^ cells were proposed to be progenitor cells and to promote regeneration of kidney tissue after ischemic injury in mice [16]. Coexpression of nestin with other markers of progenitor cells as well as their participation in tissue repair may suggest nestin as a marker of renal progenitors.

This work aimed to explore the change in the number of renal progenitor cells with age. In the kidneys of young and old transgenic nestin-GFP mice, we evaluated the number of nestin^+^ cells using total homogenates as well as histological slices. We also used the primary cultures of renal tubular cells (RTCs) obtained from the kidneys of young and old nestin-GFP mice, to study cell proliferation rate, mitochondria functioning, and the resistance of RTCs from mice of different ages to damaging stimuli, namely, oxygen–glucose deprivation (OGD) and cisplatin cytotoxicity.

## 2. Results

### 2.1. The Number of Nestin^+^ Cells in Kidneys Is Diminished with Age

**Figure 2:**
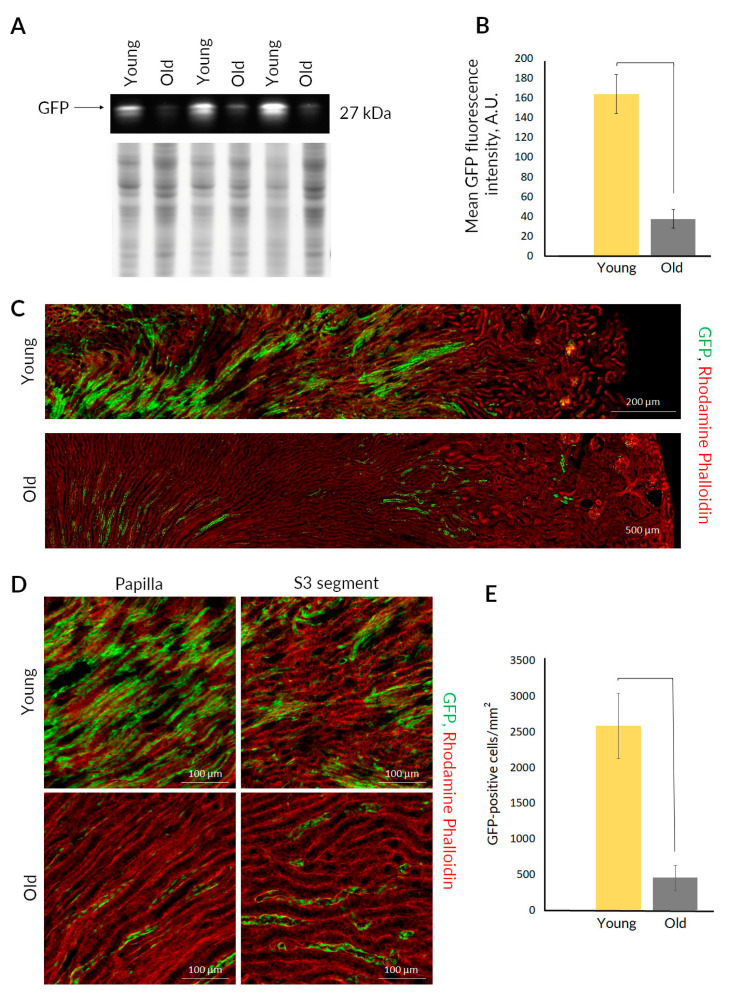
The presence and number of nestin^+^ cells in kidneys of young and old nestin-GFP mice. (**A**) GFP bands after PAGE of kidney homogenates and their normalization on total protein loading (performed by Stain-free imaging) (*n* = 3). (**B**) Normalized fluorescence intensity of GFP bands after PAGE of kidney homogenates (mean ± SD; *p*-value < 0.05 (U-test)). (**C**) Representative confocal images of kidney slices stained with rhodamine-phalloidin (*n* = 3). (**D**) Representative confocal images of S3 segment and papilla in kidney slices stained with rhodamine-phalloidin (*n* = 3). (**E**) The number of GFP-positive cells on kidney slices (mean ± SD; *p*-value < 0.05 (U-test)).

We evaluated the presence of nestin^+^ cells in the kidneys of young and old nestin-GFP mice as means of the GFP fluorescence levels in the total kidney homogenates. After polyacrylamide gel electrophoresis (PAGE), we observed that the levels of GFP fluorescence were significantly higher in the kidney from young mice (Figure 2A,B), indicating the loss of nestin^+^ cells in old animals.

We also analyzed paraformaldehyde-fixed slices of intact kidneys from young and old nestin-GFP mice (Figure 2C,D). In young mice, we detected a large number of nestin^+^ cells scattered throughout the kidney slices with a preferential location in the S3 segment and papilla areas, which were earlier proposed as possible niches for the renal resident progenitor cells [17,18]. We found a dramatic drop in the number of nestin^+^ cells in old kidney (Figure 2E). Moreover, GFP-positive tubules in old nestin-GFP mice were detected almost exclusively in the S3 segment and papilla zones. Nuclear staining with DAPI confirmed that nestin^+^ cells both in young and old mice were located in the tubules and the number of these cells was lower in kidneys of old mice (Supplementary Figure S1). The morphology of renal tissue was controlled using transmitted light imaging (Supplementary Figure S2). 

To explore the phenotype of nestin^+^ cells, the expression of Na^+^/K^+^-ATPase was analyzed using immunofluorescent staining of kidney slices from young and old nestin-GFP mice (Supplementary Figure S3). We observed that tubular epithelial cells in normal kidney displayed clear basolateral expression of Na^+^/K^+^-ATPase (Supplementary Figure S3), which is a characteristic sign of differentiated renal epithelium [19]. The localization pattern of Na^+^/K^+^-ATPase differed in the nestin^+^ tubules (Supplementary Figure S3), which may indicate a less-differentiated state of these cells. Of note, the expression of Na^+^/K^+^-ATPase in kidneys of young animals was lower compared with old ones.

### 2.2. Proliferation of RTCs Reduces with Age

**Figure 3:**
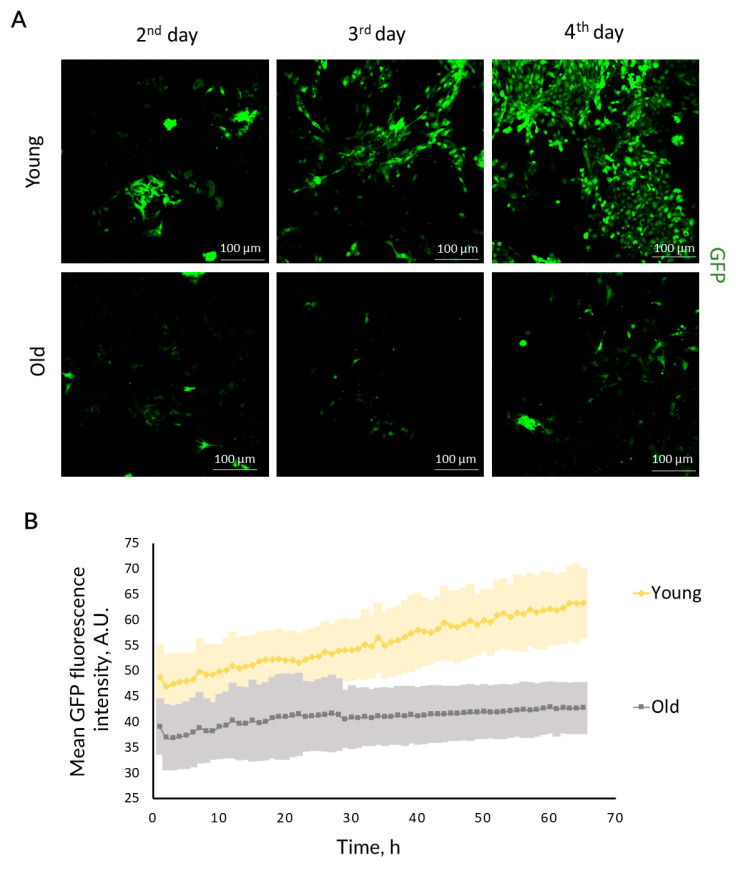
Proliferation of GFP-positive cells in the RTC cultures from young and old nestin-GFP mice. (**A**) Representative confocal images of GFP fluorescence in RTCs at the 2nd, 3rd, and 4th day of cultivation (*n* = 3). (**B**) Dynamics of total GFP fluorescence intensity in the wells with RTC during the expansion of the cultures (mean ± SD; *n* = 3).

**Figure 4:**
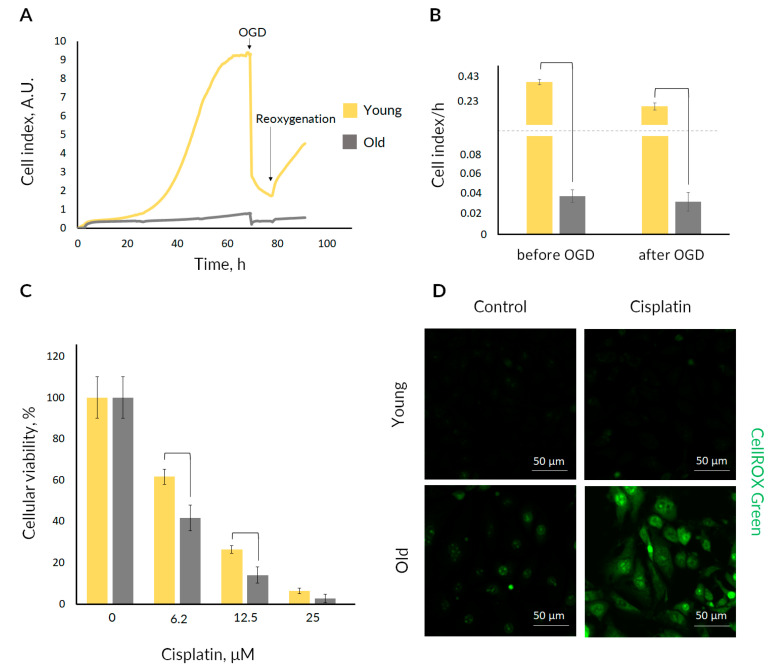
Effects of damaging factors on proliferation and viability of RTC cultures from young and old nestin-GFP mice. (**A**) The averaged growth curves of RTC cultures from young and old mice, including proliferation in normoxic conditions, cell death during OGD, and the recovery after the reoxygenation and supplementation with complete culture medium (*n* = 3). (**B**) Calculated growth rate of RTC cultures from young and old mice before and after OGD (mean ± SD; *p*-value < 0.05 (U-test)). (**C**) Cell viability of RTC cultures from young and old mice 24 h after exposure to cisplatin (mean ± SD; *p*-value < 0.05 (U-test)). (**D**) Representative confocal images of CellROX Green Reagent-loaded RTCs from young or old mice after 100 µM cisplatin treatment for 4 h (*n* = 3).

Experiments on primary RTCs revealed decreased proliferative capacity of cells from old nestin-GFP mice. When plating identical amount of kidney tubules, RTCs from young kidneys formed a confluent monolayer on the 3rd day, whereas RTCs from old mice formed a monolayer on the 5th day (Figure 3A, Supplementary Figure S4). We confirmed these observations by real-time proliferation analysis of RTCs using the iCelligence system. RTC cultures obtained from the kidneys of young mice proliferated multifold faster than the cultures from old animals (Figure 4A,B; see the growth rate before OGD).

Furthermore, we analyzed the accumulation of GFP-positive cells in the RTC cultures from nestin-GFP mice, collecting the entire GFP fluorescence intensity in the well. Fluorescence was measured for three consecutive days starting from the 2nd day of cultivation. We observed that GFP fluorescence intensity increased faster in the RTC cultures obtained from young nestin-GFP mice (Figure 3A,B), indicating higher proliferation of nestin^+^ cells than in RTC cultures from old mice.

In addition, we evaluated the proliferative characteristics of renal tissue from young and old nestin-GFP mice (Supplementary Figure S5). We observed that kidneys of young mice had higher proliferating cell nuclear antigen (PCNA) levels than old kidneys (Supplementary Figure S5A,B). We also performed immunofluorescent staining of kidney sections and showed that nuclei of some nestin^+^ cells coexpressed PCNA (Supplementary Figure S5C).

### 2.3. Resistance of RTCs to Damaging Factors Decreases with Age

We compared the effect of damaging factors, namely OGD and cisplatin cytotoxicity, on the RTCs from young and old nestin-GFP mice (Figure 4). We performed OGD with real-time monitoring of cell proliferation to assess the change of regenerative response to an injury during aging. Under normal conditions, RTCs from young mice showed a much faster growth rate than RTCs from old mice (Figure 4A,B; see the growth rate before OGD). 

OGD caused a decrease in cell index in both age groups. Real-time monitoring of RTCs proliferation revealed that after an injury caused by OGD, the recovery rate of RTCs from young mice was still much higher than that from old ones (Figure 4A,B; see the growth rate after OGD).

We also evaluated the resistance of cells from young and old mice to cisplatin cytotoxicity. Cisplatin at concentrations of 6.2 and 12.5 μM had a more substantial deterioration effect on RTC cultures from old mice expressed in diminished cellular viability. At the same concentrations of cisplatin, the survival of the RTCs from young mice was significantly higher, as measured by MTT assay (Figure 4C). We also confirmed higher cell death of old kidney cells by trypan blue assay (Supplementary Table S1); further, Annexin V-FITC staining showed that death occurred through the apoptotic pathway (Supplementary Figure S6). Taken together, these observations may indicate a greater resistance to nephrotoxic damage to RTCs from young mice compared with cells from old organisms.

Furthermore, to characterize the possible mechanism of such vulnerability of kidney cells from old animals, we analyzed the development of oxidative stress under cisplatin challenge. We found that cisplatin treatment resulted in pronounced reactive oxygen species (ROS) generation in RTCs obtained from the kidneys of old mice (Figure 4D). Such an increase in ROS production was not observed in kidney cells of young animals.

### 2.4. Mitochondrial Membrane Potential in RTCs

**Figure 5:**
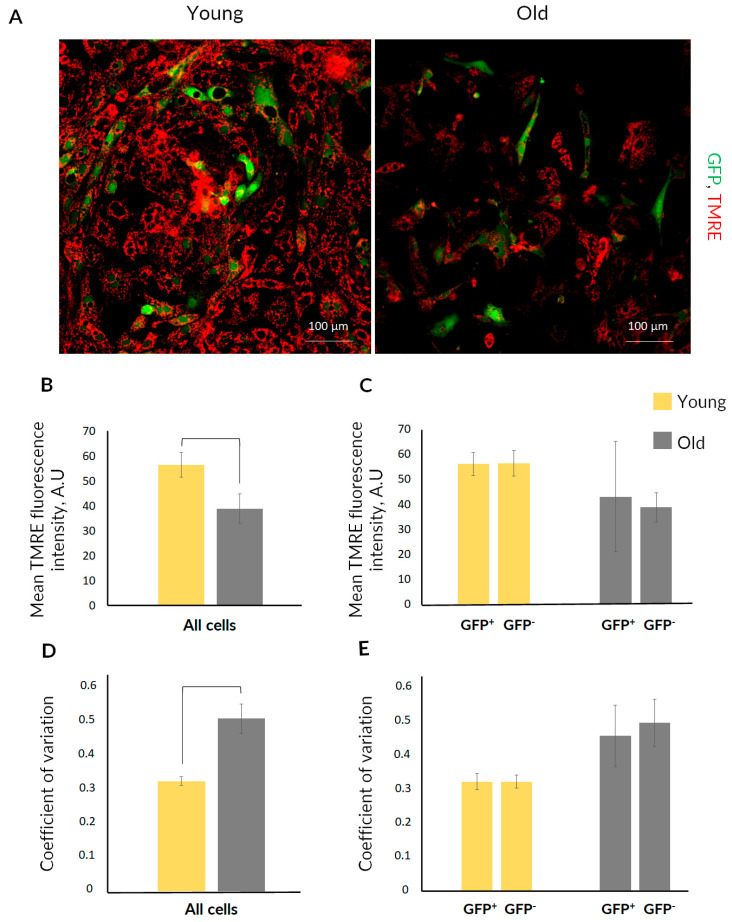
Mitochondrial membrane potential in RTC cultures from young and old nestin-GFP mice. (**A**) Representative confocal images of RTC cultures loaded with TMRE (*n* = 3). (**B**) Comparison of mitochondrial membrane potential measured by TMRE fluorescence in total RTCs population from young and old kidneys (mean ± SD; *p*-value < 0.05 (U-test)). (**C**) Mitochondrial membrane potential in the GFP-positive and GFP-negative cells in RTC cultures (mean ± SD; *p*-value < 0.05 (U-test)). (**D**) Coefficient of variation of mean TMRE fluorescence intensity in total RTCs population from young and old kidneys (mean ± SD; *p*-value < 0.05 (U-test)). (**E**) Coefficient of variation of mean TMRE fluorescence intensity in the GFP-positive and GFP-negative cells in RTC cultures (mean ± SD; *p*-value < 0.05 (U-test)).

Since mitochondrial dysfunction is one of the main hallmarks of aging, we evaluated mitochondrial transmembrane potential in the RTCs from young and old mice (Figure 5). After loading cells with mitochondrial probe TMRE, we observed that cells from the kidneys of old mice had significantly lower mitochondrial membrane potential (Figure 5A,B). We also compared the mitochondrial membrane potential in GFP-positive and GFP-negative cells from animals of both ages (Figure 5A,C). In young or old mice, we did not reveal a significant difference between mitochondrial membrane potential in GFP-positive and GFP-negative cells (Figure 5C). We also evaluated the coefficient of variation of TMRE fluorescence intensity in the total RTCs population (Figure 5D) and separately for GFP-positive and GFP-negative cells (Figure 5E). We found that mean TMRE fluorescence (i.e., mitochondrial transmembrane potential) varied more significantly in cells from old mice, indicating increased heterogeneity of mitochondria.

Low mitochondrial membrane potential in old kidney cells was accompanied by a significantly higher level of ROS production compared with the cultures from young animals (Supplementary Figure S7), indicating deterioration of mitochondria functioning during aging.

## 3. Discussion

In this study, we explored alterations of renal progenitor cells during aging. Stem cells depletion and cellular senescence were suggested to be the main hallmarks of aging [6], which have been observed in various organs and tissues [20]. Age-related changes in progenitor cells were associated with depletion of the progenitor cells pool and reduced tissue regenerative capacity. There is no consensus about the existence of resident progenitor cells in kidneys [21] and its dynamics with age, while in the other organs the situation is more evident.

Myosatellite cells, also known as resident muscle stem cells, are believed to renew muscle fibers and maintain their normal function [22]. Myosatellite cells are positively stained for specific progenitor markers (Pax7, MyF5) and retain a BrdU tag [23]. In normal muscle tissue, myosatellite cells remain in a quiescent state [24], whereas in response to injury, they are activated, start to proliferate, and form new myofibers. After regeneration is completed, myosatellite cells return to a quiescent state [23]. However, muscle fibers demonstrated a significant decrease in the ability to regenerate with age [25], which leads to the development of the common senile sign sarcopenia [26]. The depletion of the pool of resident muscle progenitor cells and the acquisition of senescence-associated phenotype were suggested to be responsible for this phenomenon [27].

Neuronal stem cells (NSCs) are considered to locate in neurogenic niches with specific microenvironments [9]. NSCs also lose their ability to self-renewal and form new neurons with age [28]. Such changes are associated with a lack of external stimuli, which is a consequence of alterations in the microenvironment of a neurogenic niche [29]. This causes NSCs’ inability to proliferate, which ultimately results in the depletion of the brain stem cells pool [30]. A consequence of these destructive changes is a reduced brain regeneration capacity and the development of various neurodegenerative senile diseases [31].

Similarly, cardiac stem cells (CSCs) play an important role in renewing heart tissue and maintaining normal organ functioning. CSCs also acquire a senescence-associated phenotype with age [8]. Being isolated from the hearts of older people, they show a lower potential for proliferation, differentiation, and clonogenicity than the young ones. These changes lead to the impaired renewal of heart tissue in the elderlies, resulting in heart failure and other age-associated heart disorders [32].

Aging-associated processes also negatively affect the gastrointestinal system, increasing the risks of various diseases and declining basal functions [10]. Such destructive changes are also associated with impaired regeneration due to the malfunctioning of intestinal stem cells (ISCs). The regeneration process is influenced by both a change in the morphology of ISCs and the architecture of their niche [33]. Interestingly, unlike other somatic stem cells, the qualitative changes in ISCs cause more pronounced impairment in their proliferative and regenerative capacity than quantitative decrease [34].

So, the depletion of the resident stem cell pool with age has been shown for many organs. However, for the kidney, there has been a long debate about the existence of resident progenitor cells [35]. Due to the great complexity of the kidney cellular organization, as well as the lack of conventional markers [36], there is still no consensus about the basis of kidney regeneration [37]. Various studies suggest two possible niches of renal progenitor cells, located mostly in the S3 segment of nephron [38] or renal papilla [39], while other studies suggest dedifferentiation of mature epithelial cells as the major mechanism of renal tissue regeneration [40]. Several studies assume that the kidney regeneration process may be mediated by mesenchymal stromal stem cells (MSCs) and paracrine regulation by their exosomes [41,42]. Therefore, the discussion about the leading pathway in renal regeneration and the origin of progenitor cells is still open.

Some studies suggest glycosylated CD133 and CD24 as markers of renal progenitor cells in human kidneys [43]. These scattered proximal tubular cells produce about 50 other proteins, which are recommended to be used as markers of these cells. CD133^+^CD24^+^ cells are also labeled with BrdU and differ in morphology; for instance, such cells have less cytoplasm and are characterized by the absence of a brush border and a small number of mitochondria. However, rodents lack these markers, which imposes the requirement to use other markers for the identification of the progenitor cells population [44].

A recent study showed that human CD133^+^ cells also co-express nestin [15]. Nestin is expressed in the neuronal progenitor cells, in the germ skeletal and cardiac myocytes, and in the mesonephros and endothelial cells of developing vessels [14]. Therefore, nestin is suggested to be a marker of progenitor cells in a broad spectrum of organs, and this protein is widely expressed during kidney development [45]. In the kidneys, nestin^+^ cells also co-express other markers of progenitor cells (including Flk-1, Tie-2, CD34, CD150) [16]. Renal nestin^+^ cells are characterized by label-retaining, which indicates their slow cell cycle [17]. Following renal damage, nestin expression was induced in renal tubule cells and glomerular mesangial cells, indicating its role in tissue repair [46,47]. In particular, recent study showed that nestin^+^ cells displayed medullary-to-cortical migration after kidney ischemia, which may be interpreted as its participation in the regeneration of injured nephrons [16]. 

Earlier, to determine whether the presence of GFP fluorescence correlated with nestin expression, the distribution of GFP-positive cells was compared with the distribution of nestin-immunoreactive cells [13,16]. Such analysis showed that the pattern of GFP-positive and nestin^+^ cells was similar in the kidneys and brain of nestin-GFP mice, which allowed us to use GFP fluorescence as a marker of nestin expression in our experiments. GFP half-life is relatively short (about 26 h) [48], which limits the probability of GFP presence in the differentiated daughter cells. Of note, for kidneys, the constitutive expression of nestin by mature podocytes must be considered [49], including in nestin-GFP-transgenic mice.

Considering the above arguments, we used transgenic nestin-GFP mice to evaluate the change in progenitor cells number in kidneys during aging. In kidney slices and total tissue homogenates, we observed a decrease in the number of nestin^+^ cells in kidneys of old nestin-GFP mice compared with young animals (Figure 2). It is important that the histological examination of kidneys from both young and old nestin-GFP mice revealed an exclusive localization of nestin^+^ cells in their putative niches (Figure 2C,D), the S3 segment and the papilla [21]. We also demonstrated that nestin^+^ cells have some characteristic features of progenitor cells, including different patterns of Na^+^/K^+^-ATPase localization (Supplementary Figure S3), which is a marker of mature epithelial cells [19]. Moreover, nuclei of some nestin^+^ cells expressed PCNA (Supplementary Figure S5), the marker of proliferating cells [50]. 

In vitro experiments on the primary RTC cultures demonstrated that RTCs obtained from the kidneys of old mice had a reduced content of nestin^+^ cells (Figure 3) and a much lower proliferation rate (Figure 4A, see growth before OGD), indicating the decrease in the number of proliferating cells and/or their proliferative capacity. Analysis of the GFP levels during RTCs expansion showed that cultures from young animals were characterized by a faster increase in GFP intensity (Figure 3). 

Moreover, we demonstrated that cells from old mice were less resistant to ischemic and nephrotoxic damaging stimuli compared with RTCs from young animals (Figure 4, Supplementary Table S1, Supplementary Figure S6). Similar to stem cells in other organs, renal progenitor cells may also undergo adverse age-related changes, which may affect their response to a challenge [26,51]. It is well described that aged organisms are more susceptible to kidney injury and organ recovery is much worse than in a young organism [52,53]. Cisplatin was also previously shown to cause a stronger nephrotoxic side effect on the aged kidney [54]. Such a deleterious effect is supposed to be associated with more pronounced oxidative stress induced by cisplatin in the cells of aged kidney and excessive accumulation of the drug. We also found that cisplatin treatment results in more pronounced oxidative stress in cells isolated from the kidneys of old mice (Figure 4D).

Another hallmark of aging is mitochondrial dysfunction and the progressive increase in mitochondrial heterogeneity [6,55]. Analysis of mitochondrial membrane potential in the RTC cultures from young and old mice revealed the drop in mitochondrial potential in cells from old animals (Figure 5A,B). Earlier studies described bioenergetics impairment in the various tissues of aged organisms; in particular, the mitochondrial membrane potential was significantly lower probably due to higher proton leak [56]. We analyzed mitochondrial heterogeneity in kidney cells during aging. High mitochondrial heterogeneity in terms of the transmembrane potential may reflect deleterious changes in mitochondria during pathological conditions and aging [57,58]. We showed that RTCs from old mice, besides low membrane potential, had significantly higher mitochondrial heterogeneity compared with cultures from young animals (Figure 5D), indicating the accumulation of poorly functioning mitochondria.

There is still no consensus on whether the mitochondrial membrane potential in stem cells differs from that in the differentiated cells. For instance, hematopoietic stem and progenitor cells demonstrate an increased mitochondrial membrane potential compared with differentiated cells [59]. On the other hand, mitochondria in induced pluripotent stem cells were found to lack well-defined cristae and have decreased mitochondria function [60]. We compared the mitochondrial membrane potential in nestin^+^ and nestin^−^ cells of RTCs culture obtained from young and old nestin-GFP mice to estimate mitochondrial changes in progenitor and non-progenitor cells (Figure 5C,E). The analysis did not show significant differences in the mitochondrial membrane potential between nestin^+^ and nestin^−^ cells, in kidneys both from young and old mice. However, as mentioned above, mitochondrial functions decreased in old mice in both nestin^+^ and nestin^−^ cells. 

Impaired mitochondrial functioning and resulting changes in mitochondrial membrane potential being a typical feature of cells from old organisms, which may also cause and regulate the level of oxidative stress [61]. Excessive ROS content is considered to be one of the hallmarks of aging [62]. Increased oxidative damage was observed in the renal cells of elderly organisms [63,64]. Indeed, cells from old mice demonstrated higher levels of ROS production (Supplementary Figure S7). Oxidative stress is suggested to make a significant contribution to the decline in renal function with age and increased kidney susceptibility to injury [65,66]. Improper functioning of the antioxidant system is believed to be one for the reasons of such elevation in ROS levels during aging [67,68]. The question of whether stem cells differ from differentiated cells in ROS production and oxidative stress needs further research. 

## 4. Materials and Methods

### 4.1. Animals

The study was performed on the male nestin-GFP transgenic reporter mouse strain [13]. The nestin-GFP mice were generated [69] and kindly provided by Grigori Enikolopov. The experiments were carried out on homozygous transgenic mice of two ages (Figure 1): young 21-day-old mice and aged 1-year-old mice. The age of old nestin-GFP mice was chosen due to the early manifestation of signs of aging in the mice of this strain. Some experiments were performed on wild-type BALB/c mice of the same age to avoid cross-talk of fluorescent probes with GFP fluorescence. Animal protocols were evaluated and approved by the animal ethics committee of the A.N. Belozersky Institute of Physico-Chemical Biology Lomonosov Moscow State University: Protocol 3/19 from 18 March 2019. All procedures were in accordance with the “Animal Research: Reporting of In Vivo Experiments” (ARRIVE) guidelines. The animals had unlimited access to food and water and were kept in cages in a temperature-controlled environment (20 ± 1 °C) under a 12/12 h light/dark regime. The overall number of animals in each age group was 15. 

### 4.2. PAGE

GFP levels were analyzed using PAGE of the kidney tissue taken from young (*n* = 3) and old (*n* = 3) nestin-GFP mice. Mice were euthanized, and kidneys were isolated and homogenized with a glass-Teflon homogenizer in a PBS buffer containing 1 mM phenylmethylsulfonylfluoride at 4 °C. The homogenates were centrifuged at 3000× *g* for 3 min; the supernatants were mixed with 4× sample buffer containing 10% 2-mercaptoethanol without further boiling. The total protein concentration in the samples was measured using a bicinchoninic acid assay (Sigma, Burlington, MA, USA). For electrophoresis, kidney samples were loaded onto a gradient 10%–20% Tris-glycine polyacrylamide gel with total protein concentration 10 μg/lane. After electrophoresis, the detection of GFP fluorescence in the gel was performed by a V3 Western Blot Imager (BioRad, Hercules, CA, USA) at the excitation wavelength of 488 nm. Total protein loading was controlled by Stain-free imaging technique according to the manufacturer’s instructions (#1610185, BioRad, Hercules, CA, USA).

### 4.3. Western Blotting

Mice (*n* = 3 for each group) were euthanized, and kidneys were isolated and homogenized with a glass-Teflon homogenizer in a PBS buffer containing 1 mM phenylmethylsulfonylfluoride at 4 °C. The homogenates were centrifuged at 3000× *g* for 3 min; the supernatants were mixed with 4× sample buffer containing 10% 2-mercaptoethanol and boiled for 5 min. The total protein concentration in the samples was measured using a bicinchoninic acid assay (Sigma, USA). Kidney samples were loaded onto 10%–20% Tris-glycine polyacrylamide gels (10 μg protein/lane). After electrophoresis, gels were transferred onto PVDF membranes (Amersham Pharmacia Biotech, Buckinghamshire, UK). Membranes were blocked with 5% non-fat milk in PBS with 0.05% Tween-20 and subsequently incubated with primary antibodies: anti-PCNA 1:1000 rabbit (#13110, Cell Signaling, Danvers, MA, USA), anti-β-actin 1:2000 mouse (#A2228, Sigma-Aldrich, St. Louis, MO, USA). Membranes were then incubated with secondary antibodies, anti-rabbit IgG or anti-mouse IgG conjugated with horseradish peroxidase 1:7500 (Jackson ImmunoResearch, West Grove, PA, USA), and probed with Advansta Western Bright ECL kit (Advansta, San Jose, CA, USA). Detection was performed by a V3 Western Blot Imager (BioRad, USA).

### 4.4. Histological Study 

Paraformaldehyde-fixed kidneys (*n* = 3 in each age group) were incubated in 10%–20%–30% sucrose; then, they were placed in a cryoembedding medium (Tissue-Tek^®^ O.C.T. Compound, Tokyo, Japan) and frozen in liquid nitrogen vapor. Using a cryotome (Leica, Wetzlar, Germany), 10 µm cryosections were made and placed onto glass slides coated with polylysine (ThermoFisher Scientific, Erlangen, Germany). The cryoembedding medium was removed, and slices were permeabilized in PBS containing 1% bovine serum with 0.1% Triton X-100 for 30 min at room temperature. 

For actin staining, slices were incubated with 1 μM rhodamine-phalloidin (R145, Invitrogen, Waltham, MA, USA) in PBS with 0.1% Triton X-100 overnight at 4 °C. After incubation, slices were washed out three times for 5 min in PBS with 0.1% Triton X-100 and placed in a Fluoroshield Mounting Medium (Sigma, USA). 

For immunofluorescent staining, sections were incubated with primary antibodies against Na^+^/K^+^-ATPase (ab7671, Abcam, Cambridge, UK) or PCNA (13110, Cell Signaling, USA) overnight at 4 °C. Then, the slices were washed 3 times for 5 min with PBS and 0.1% Triton X-100 and incubated for 1 h with secondary antibodies: Cy5-conjugated goat anti-mouse (115-175-146, Jackson ImmunoResearch Laboratories Inc., Cambridge, UK) or Cy5-conjugated goat anti-rabbit (111-175-144, Jackson ImmunoResearch Laboratories Inc., UK). The slices were washed 3 times for 5 min with PBS with 0.1% Triton X-100 and incubated with 300 nM DAPI (D1306, Invitrogen, ThermoFisher Scientific, Waltham, MA, USA) for 20 min in PBS with 0.1% Triton X-100. After 3 times washing for 5 min with PBS with 0.1% Triton X-100, slices were mounted with Fluoroshield mounting medium (Sigma, USA).

After staining, slices were analyzed with LSM 900 inverted confocal microscope (Carl Zeiss, Oberkochen, Germany). GFP fluorescence was recorded at 488 nm excitation and emission at 500–530 nm. Rhodamine-phalloidin and Cy5 fluorescence was evaluated using a 543 nm excitation wavelength with an emission >616 nm. DAPI was excited at 405 nm with emission collected at 410–483 nm. For all slices, we also performed transmitted light imaging. The images were analyzed using Fiji/ImageJ open-source software (version 2.9.0) [70].

### 4.5. Culture of RTCs

For in vitro experiments, primary cultures of RTCs were isolated from the kidneys of young and old nestin-GFP mice. The mice were euthanized, and kidneys were aseptically isolated, cut into small pieces, and incubated with 0.25% collagenase II type (Gibco, ThermoFisher Scientific, USA) in DMEM/F12 bicarbonate-free media at 37 °C for 15 min for young mice and 30 min for old mice. Kidney pieces were pipetted, and the resulting suspension was centrifuged for 5 min at 400× *g* to pellet the tubular fraction. The pellet was resuspended in a complete DMEM/F12 culture medium with 10% fetal bovine serum (FBS). The resulting renal tubules were plated on cultural dishes. After 48 h, the medium was changed to remove cellular debris. RTCs obtained from young mice formed a monolayer approximately on the 3rd day of cultivation, whereas the RTCs from kidneys of old mice formed a monolayer on the 5th day; so, the cultures were analyzed after 2–5 days of cultivation. Cell count was performed using transmitted light imaging on an LSM 900 confocal microscope.

### 4.6. Dynamics of GFP Levels 

GFP fluorescence intensity in RTC cultures (*n* = 3 in each age group) was assessed on the 2nd, 3rd, and 4th day of cultivation. For these experiments, the cultures of RTCs were plated on FluoroDish (WPI, Worcester, MA, USA). GFP fluorescence detection was carried out using an LSM 900 confocal microscope at 488 nm excitation and emission 500–530 nm. The images were analyzed using the Fiji/ImageJ software.

GFP changes were confirmed using a ClarioStar microplate reader (BMG Labtech, Ortenberg, Germany). RTC cultures from nestin-GFP mice were plated on 96-well plates. On the 2nd day of cultivation, the complete culture media was replaced by phenol-red-free culture media. Plates were analyzed in the ClarioStar reader for three consecutive days, measuring GFP fluorescence intensity every 1.5 h.

### 4.7. OGD

The cell proliferation was estimated using the iCelligence real-time cell analysis system (Agilent Technologies, Santa Clara, CA, USA). RTCs (*n* = 3 in each age group) were cultured in special 8-well plates for the iCelligence system. On the 3rd day, the cells were subjected to OGD, during which the complete culture medium was replaced by the sterile DPBS solution and put into a Galaxy 170R multigas incubator (Eppendorf/NewBrunswick, Hamburg, Germany) with 1% O_2_ and 0% CO_2_ for 6 h. Then, the normoxic complete cultural medium was returned, initiating reoxygenation.

### 4.8. MTT Assay

Cisplatin cytotoxicity was studied in vitro using RTC cultures (*n* = 3 in each age group). The viability of RTC cultures from old and young nestin-GFP mice after exposure to nephrotoxic cisplatin was measured by MTT assay. RTCs were cultured in 96-well plates for 4 days; then, separately, 6.2 μM, 12.5 μM, 25 μM, and 50 μM of cisplatin were added to the cells for 24 h. The standard MTT assay was carried out, and the absorption was measured at 540 nm using a Zenyth 3100 plate multimode detector (Anthos Labtec, Wals, Austria). Wells with cells incubated with H_2_O for 24 h were used as a negative control. 

### 4.9. Trypan Blue Assay

To evaluate the number of live and dead cells in the RTC cultures (*n* = 3 in each age group) after cisplatin treatment, we used trypan blue assay. RTCs were dissociated by 0.25% trypsin-EDTA; cells were loaded with 0.4% trypan blue dye (Gibco, ThermoFisher Scientific, USA) at the ratio 1:1. Counting of live and dead cells was performed using a Luna™ cell counter (Logos Biosystems, Annandale, VA, USA).

### 4.10. Annexin V Staining

Annexin V-FITC staining (Invitrogen, ThermoFisher Scientific, USA) was used to estimate apoptosis induction in RTC cultures from young and old mice after 6.2 µM cisplatin treatment for 24 h (*n* = 3 in each age group). Annexin V-FITC was prepared according to manufacturer’s instructions and incubated with RTCs for 15 min. Annexin V-positive cells were analyzed using an LSM 900 confocal microscope with 488 nm excitation wavelength and emission collected at 500–530 nm.

### 4.11. CellROX Green Staining

To assess the ROS production, RTCs were loaded with 5 µM CellROX Green reagent (Invitrogen, ThermoFisher Scientific, USA) for 30 min at 37 °C (*n* = 3 in each age group). CellROX Green fluorescence was evaluated using 488 nm excitation wavelength with emission collected at 500–530 nm using an LSM 900 confocal microscope.

### 4.12. TMRE Staining 

To assess the mitochondrial membrane potential, RTCs (*n* = 3 in each age group) were loaded with 200 nM tetramethyl-rhodamine ethyl ester (TMRE) for 30 min at 37 °C. TMRE and GFP fluorescence were evaluated using 543 nm and 488 nm excitation wavelengths with emission collected at >616 nm and 500–530 nm, correspondingly using an LSM 900 confocal microscope. Image analysis was performed using Fiji/ImageJ software.

### 4.13. Statistical Analysis

Data processing and statistical analysis were performed using Microsoft Excel 2016 (version KB4011684, Redmond, WA, USA). The results were expressed as mean ± SD. Comparisons between groups were made by Mann–Whitney U test (*p*-value < 0.05).

## 5. Conclusions

The depletion of the resident progenitor cell pool with age has not been sufficiently investigated for kidney tissue yet. In this study, we evaluate the change in the number of renal progenitor cells with age using the model of nestin-GFP transgenic reporter mice. We found a dramatic decrease in the number of nestin^+^ renal resident progenitor cells in the kidneys of old mice associated with lower proliferation rate of the cells from old animals and diminished tolerance to the damaging factors. We suggest that reduced regenerative potential of the aged kidney may be explained by the exhaustion and dysfunction of the renal resident progenitor cell pool.

## Data Availability

The data that support the findings of this study are available from the corresponding author upon reasonable request.

## Supplementary Materials

The following supporting information can be downloaded at: https://www.mdpi.com/article/10.3390/ijms231911015/s1.

## Abbreviations

AKI—acute kidney injury; CKD—chronic kidney disease; CSCs—cardiac stem cells; ISCs—intestinal stem cells; NSCs—neuronal stem cells; FBS—fetal bovine serum; GFP—green fluorescent protein; OGD—oxygen–glucose deprivation; PAGE—polyacrylamide gel electrophoresis; PCNA—proliferating cell nuclear antigen; RTCs—renal tubular cells; ROS—reactive oxygen species; TMRE—tetramethyl-rhodamine ethyl ester.
