# Supplementary material for: Age-Associated Loss in Renal Nestin-Positive Progenitor Cells
Source: Int J Mol Sci. 2022 Sep 20;23(19):11015. doi: 10.3390/ijms231911015 (PMC9569966; doi:10.3390/ijms231911015)
Supplement: Supplementary file 1 [file ijms-23-11015-s001.zip › ijms-1889464-supplementary.pdf]

Age-associated loss in renal nestin-positive progenitor cells

Marina I. Buyan, Nadezda V. Andrianova, Vasily A. Popkov, Ljubava D. Zorova, Irina B. Pevzner, Denis N. Silachev, Dmitry B. Zorov and Egor Y. Plotnikov

Supplementary Figures

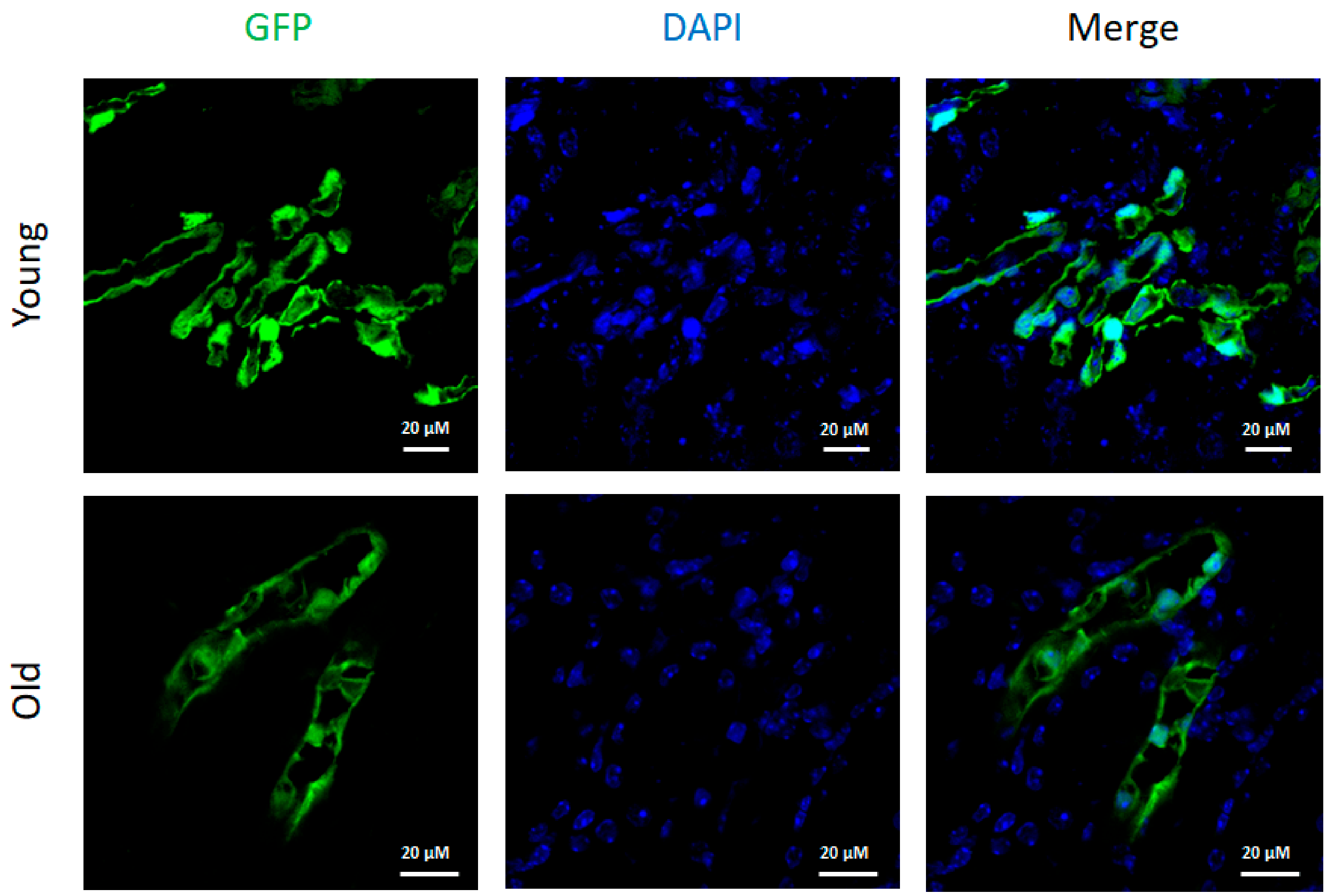

Supplementary Figure S1. DAPI (blue) staining of nuclei in kidney slices from young and old nestin-GFP mice.

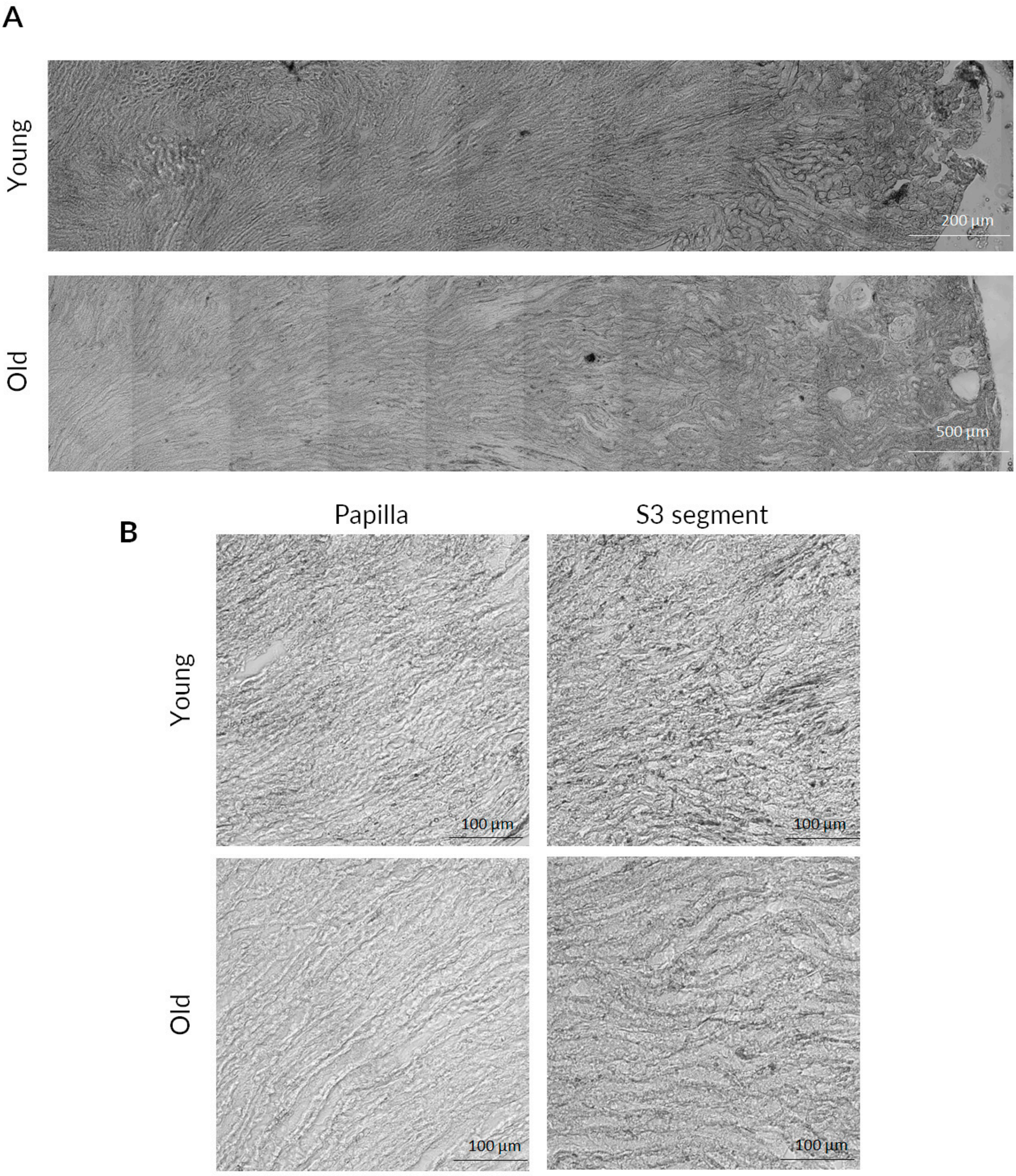

Supplementary Figure S2. Transmitted light images of kidney slices from young and old nestin-GFP mice. **(A)** Representative panoramic images of kidney slices; **(B)** Representative images of S3 segment and papilla.

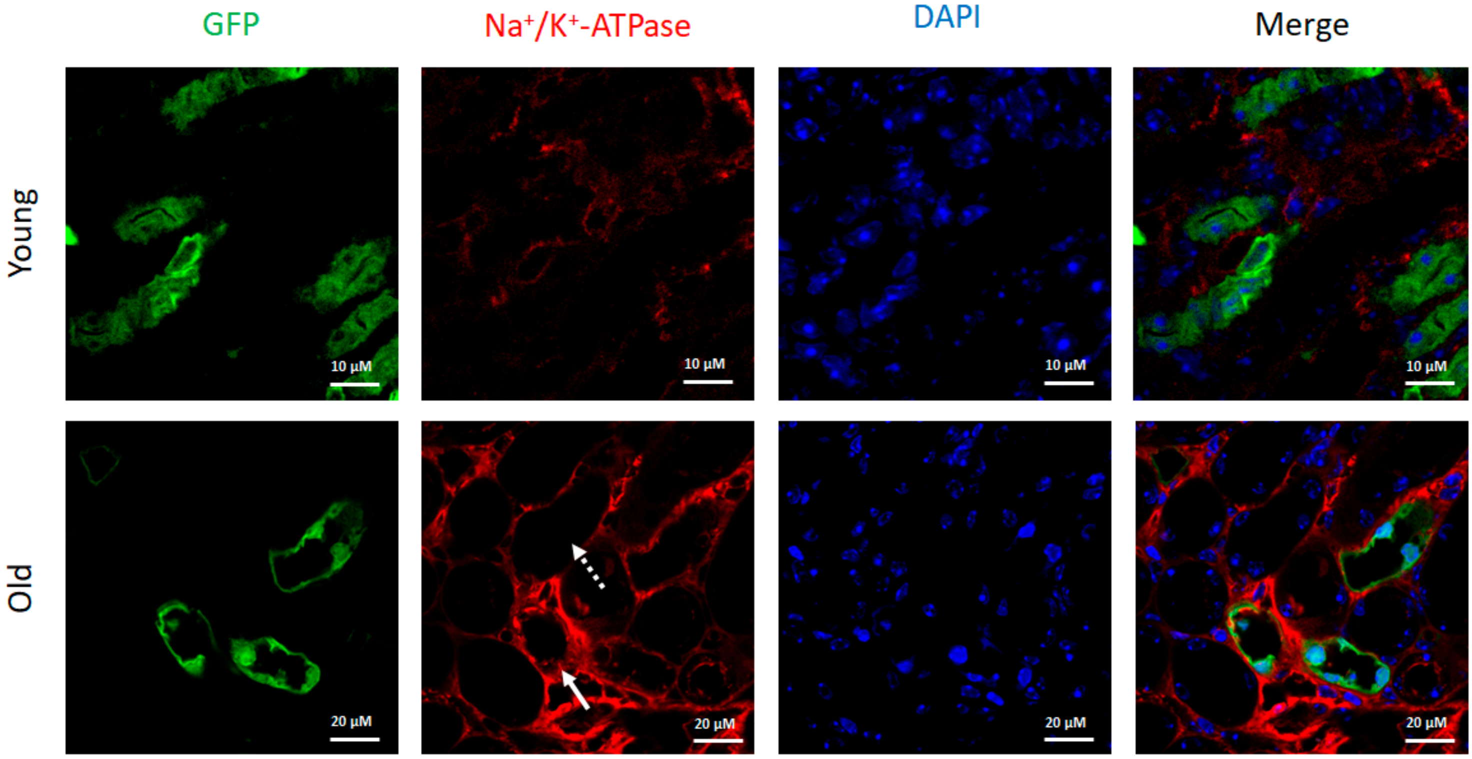

Supplementary Figure S3. Immunofluorescent staining with antibodies against Na^+/K^+-ATPase of kidney slices from young and old nestin-GFP mice (solid white arrow indicates Na^+/K^+-ATPase expression in $nestin^+$ cells; dotted white arrow shows Na^+/K^+-ATPase expression in $nestin^-$ cells).

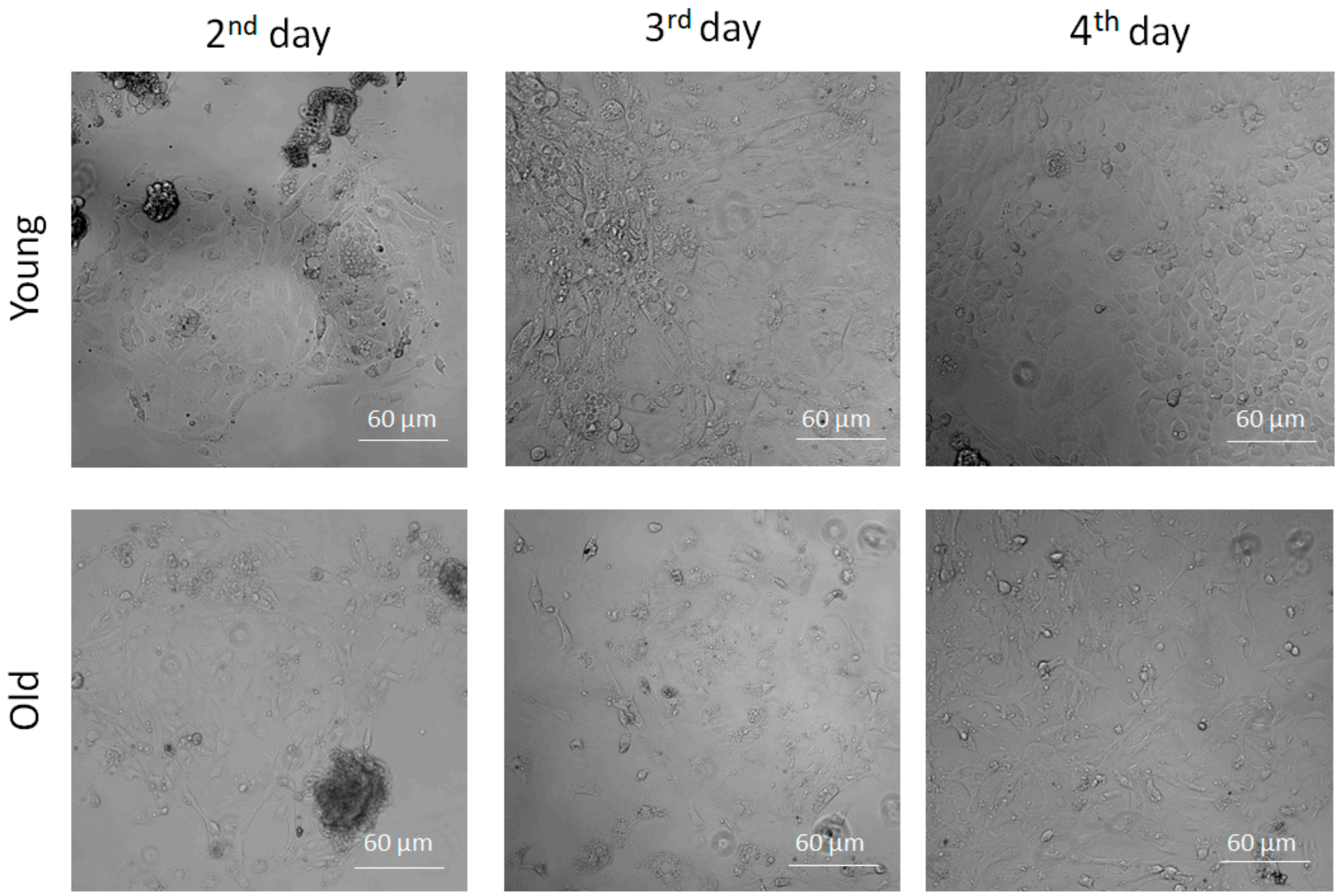

Supplementary Figure S4. Transmitted light images of RTCs from young and old nestin-GFP mice during their growth.

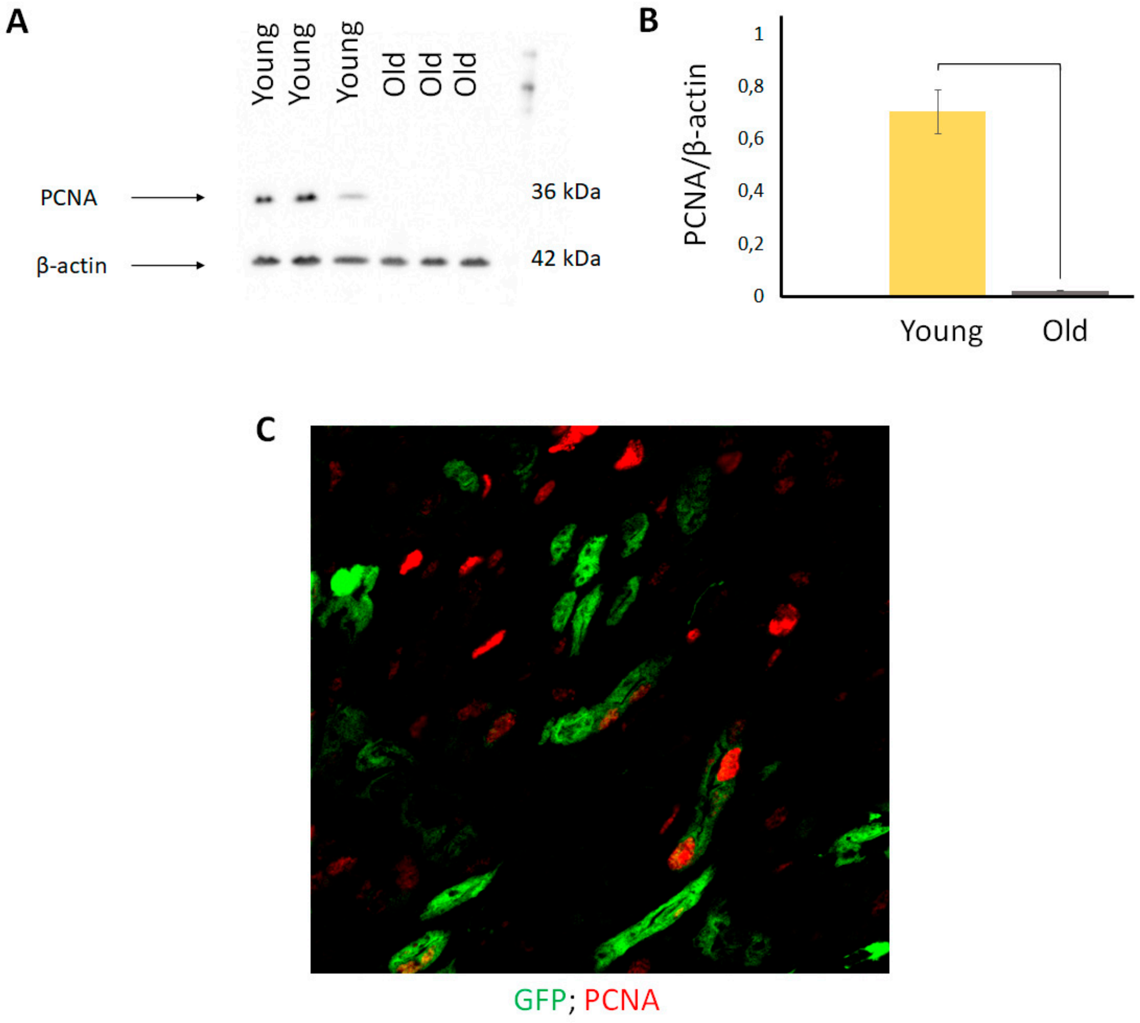

Supplementary Figure S5. Evaluation of PCNA-positive cells in the kidneys from young and old transgenic mice. **(A, B)** PCNA levels in kidney homogenates of young and old transgenic mice (mean ± SD; P-value < 0.05 (U-test)); **(C)** Representative confocal image of kidney slices from young nestin-GFP mice stained with antibodies against PCNA.

Supplementary Table S1. Live and dead cell concentration and viability of RTCs from young and old mice with 6.2 μM cisplatin treatment for 24 h.

Age	Live cell concentration (cells/mL)	Dead cell concentration (cells/mL)	Viability, %
Young (control)	5.92×10^5	1.30×10^5	82.1
Young (cisplatin)	4.91×10^5	2.50×10^5	66.2
Old (control)	2.92×10^5	2.68×10^5	52.1
Old (cisplatin)	2.73×10^5	4.21×10^5	39.3

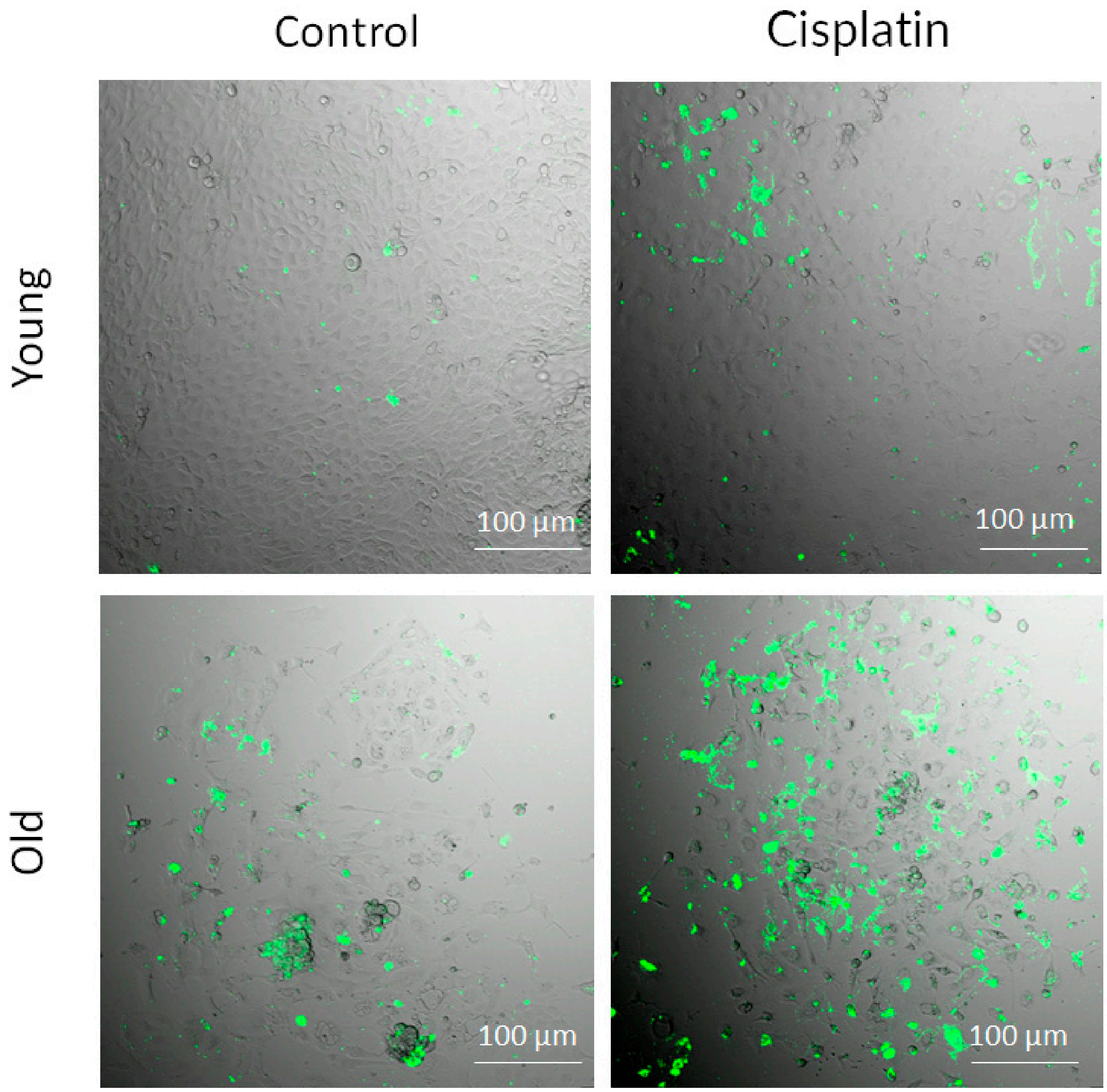

Supplementary Figure S6. Representative confocal images of Annexin V-FITC staining of RTCs cultures from young and old mice after 6.2 µM cisplatin treatment for 24 h.

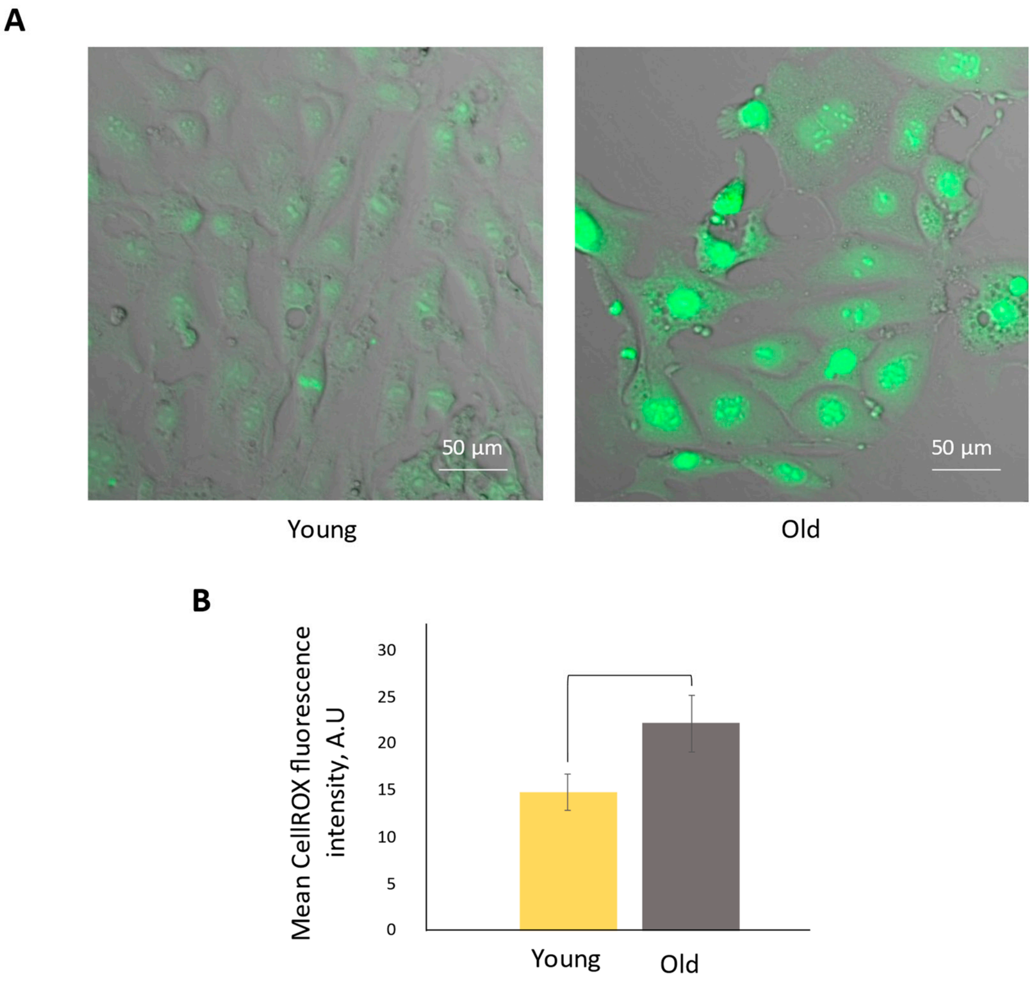

Supplementary Figure S7. Oxidative stress in RTCs cultures from young and old mice. **(A)** Representative confocal images of cells loaded with CellROX; **(B)** Mean fluorescence intensity of cells after CellROX staining (mean ± SD; P-value < 0.05 (U-test)).
